# The Predictive Value of Red Cell Distribution Width in End-Stage Colorectal Cancers’ 6-Month Palliative Chemotherapy Response—A Single Center’s Experience

**DOI:** 10.3390/jpm15080359

**Published:** 2025-08-07

**Authors:** Maciej Jankowski, Krystyna Bratos, Joanna Wawer, Tomasz Urbanowicz

**Affiliations:** 1 Clinical Oncology and Immuno-Oncology Department with Day Outpatient Sub-Department and Reception Unit of Greater Poland Cancer Centre, 61-866 Poznan, Poland; 2 Department of Experimental Immunology, Medical University of Lublin, Chodźki 4a Street, 20-093 Lublin, Poland; 3 Cardiac Surgery and Transplantology Department, Poznan University of Medical Sciences, 61-848 Poznan, Poland

**Keywords:** GIC, four-stage cancer, chemotherapy, male sex

## Abstract

**Backgrounds:** The incidence of gastrointestinal cancers (GICs), though decreased in recent years, still accounts for 35% of all cancer-related mortality. The proper identification of risk factors, early diagnosis, and therapy optimization represent the three cornerstones of GIC treatment. In four-stage diseases, chemotherapy embodies target therapy that may prolong patients’ expectancy when suitably applied. **Patients and Methods:** There were 133 (82 (62%) male and 51 (38%) female) consecutive patients with a median age of 70 (64–74) years who underwent palliative treatment due to four-stage colorectal cancer (CRC) between 2022 and 2024. The demographic, clinical, and laboratory data and applied chemotherapeutic protocols were evaluated regarding the response to applied therapy, resulting in complete or partial tumor regression. The advancement of the tumor was based on computed tomography (CT) performed before and 6 months after the chemotherapy. **Results:** The multivariable model revealed red cell distribution width (RDW) from peripheral blood analysis (OR: 0.81, 95% CI: 0.65–1.00, *p* = 0.049) as a possible predictor for systemic treatment response in colorectal cancer. The receiver operating characteristic curve revealed a predictive value of male sex and RDW prior to systemic therapy, with an area under the curve of 0.672, yielding a sensitivity of 70.0% and specificity of 58.1%. **Conclusions:** The results of our analysis point out the possible modulatory impact of RDW on six-month systemic therapy in colorectal terminal cancer management. Further studies are required to confirm the presented results.

## 1. Introduction

The incidence of gastrointestinal cancers (GIC) has been reported to have decreased in recent years, still accounting for 26% of the overall cancer prevalence and 35% of all cancer-related mortality [1]. In particular, gastric, esophageal, and biliary tract cancer incidence, when standardized to patients’ age, has been reduced in recent years; the opposite trend is being observed regarding liver, colorectal, and pancreatic disease [2]. The worldwide lifetime risk of GIC presents disparities across countries and demands distinct approaches in specific regions [3]. Dietary factors (such as alcoholic consumption and nutrient deficiencies) and non-dietary factors (such as tobacco use and certain infections) are associated with carcinogenesis [4].

Colorectal cancer (CRC) is one of the major epidemiological oncological problems worldwide [5]. Despite a growing body of research focused on early diagnosis and risk factor identification, late onset is still a medical challenges associated with inferior prognosis [6]. CRC represents one of the most common malignancies with established risk factors such as older age, male predominance, hereditary predisposition, physical inactivity, and excessive adipose tissue accumulation [7]. The analysis of the GLOBOCAN database [8] indicates an increasing trend of younger age among CRC patients and confirms a higher risk for postoperative complications but not increased mortality risk in older individuals. In the stage T4 colorectal cancer population, age did not impact patients’ survival according to Osseis et al. analysis [9].

Up to one out of ten new CRC cases are linked to non-polyposis colorectal cancer, familial adenomatous polyposis, or other hereditary syndromes [10].

A sex-related imbalance in pathophysiological conditions has been postulated, suggesting a higher prevalence of autoimmune diseases in females and cancerous risk in the male population secondary to lifestyle, oncogenic viruses, and sex hormones [11]. Food additives are postulated to possess potential effects on intestinal cellular functions and gut inflammation by increasing the inflammatory cells, resulting in macrophage activation [12]. A risk score for digestive tumor development has been created [13] based on the following elements: gender, alcohol consumption, tobacco use, obesity, family history, and unhealthy diet. Launching adjusted preventive programs is one of the primary targets.

The second goal relates to early diagnosis approaches. Cancer is regarded as a complex disease involving abnormal transformation into tumor cells. The hereditary and lifestyle-related factors that predispose individuals to tumor formation and possible markers related to abnormal cells, their precancerous forms, and cancer cells can be considered biomarkers for early detection, prognosis determination, or therapy prediction [14]. Among possible markers, peripheral blood indices have attracted clinical attention in oncology due to their accessibility and cost-effectiveness [15,16,17,18].

The third step relies on therapy optimization. Surgical resection is regarded as the gold-standard therapeutic approach for localized tumors, though the relapse and progression risks are substantial. Targeted therapies for advanced stages have been developed [19]; these range from immunotherapy to standard palliative chemotherapy for improved outcomes [20,21]. Still, new therapies are under development, and understanding possible risks influencing outcomes is among the most important clinical goals.

Based on computed tomography results, this study aimed to point out possible predictors of response to six months of systemic palliative therapy in end-stage colorectal cancers.

## 2. Materials and Methods

A retrospective analysis was performed on consecutive patients with advanced-stage colorectal cancer who underwent first-line palliative treatment between 2022 and 2024 in the Clinical Oncology and Immuno-Oncology Department within the Day Outpatient Sub-Department and the Reception Unit of Greater Poland Cancer Centre. Chemotherapy results based on computed tomography imaging within 6 months after the initial systemic protocol were evaluated in the analysis. The time interval between the diagnosis and administration of systemic therapy was within the standard clinical care gap, reaching 2–4 months. Patients on a restrictive diet and those reporting alcohol use were excluded from the analysis. Nutritional status was taken into consideration, as cachexia was considered an exclusion criterion. None of the patients presented active infection episodes during enrollment or during the control hospitalization. As the aim of the analysis was to verify the effectiveness of six months’ computed tomography results, only patients with initial systemic therapy that was not modified within the 6-month period were included in the analysis.

Patients were treated with chemotherapy (FOLFOX, FOLFIRI, CAPECTABINE, FLUOROURACIL OR IRINOTECAN) protocol (cth) (47 (57%) patients) or chemotherapy with a-EGFR (anti-epidermal growth factor receptor) (cetuximab or panitumumab) (36 (43%) patients) for colorectal cancer in a palliative care setting. The exclusion criteria included patients with previous chemotherapy due to palliative colorectal cancer or patients with modified chemotherapy after a three-month course, followed by control computed tomography results.

### 2.1. Methods

The demographic, clinical, and laboratory data and applied chemotherapeutic protocols were evaluated regarding the response to applied therapy, resulting in complete or partial tumor regression.

The advancement of the tumor was based on computed tomography (CT) performed before and 6 months after the chemotherapy. CT imaging was performed using a standard protocol. The examinations were performed according to the same protocol, using two scanners: the GE Optima CT660 and Siemens Somatom Definition AS. The scans were performed based on clinical indications. Both scanners produced axial images with 1.25 mm slice thickness and a soft tissue reconstruction kernel. The same experienced team of radiologists examined the images.

Patients were grouped based on CT imaging results into responders (partial or complete response) and non-responders (stable disease or progression).

### 2.2. Statistical Analysis

Since the data did not follow a normal distribution, continuous variables were reported as medians and interquartile ranges (Q1–Q3). Categorical data were presented as numbers and percentages. The Mann–Whitney test compared interval parameters between proximal and non-proximal groups. Categorical data were compared using a chi-square test of independence. Both univariate and multivariable models were used to predict the efficiency of chemotherapy protocols based on CT imaging. The multivariable model was assessed using the best subset method. The results were presented as odds ratios (ORs) and 95% confidence intervals (95% CIs). The receiver operator curve (ROC) was used to check the accuracy of confirming multivariable analysis results in the prediction model.

### 2.3. Bioethics Committee

The study was conducted in accordance with the Declaration of Helsinki and approved by the Ethics Committee of Poznan University of Medical Sciences, Poznan, Poland (protocol code 405/24 from 19 June 2024) for studies involving humans. The patients’ consent was waived due to the retrospective nature of the analysis.

## 3. Results

There were 83 (52 (63%) male and 20 (37%) female) consecutive patients with a median age of 69 (64–74) years who underwent first-line palliative treatment due to advanced stage colorectal cancer between 2022 and 2024. There were no fatal events in the analyzed group throughout the six-month period of chemotherapy. The analysis was based on patients whose therapy was not changed or modified due to side effects throughout the analysis period.

On CT scan images, complete response, partial response, stable disease, and disease progression were observed in 2 (2%), 38 (46%), 36 (44%), and 7 (8%) patients, respectively. Patients were compared between the subgroups formed depending on therapy response in the primary analytic step. There were no significant differences in demographic and clinical data between the four categories (complete response, partial response, stable disease, and disease progression). Detailed characterization of the patients in relation to 6 months’ computed tomography results is presented in Table 1.

In the second step, patients were grouped based on CT imaging results into responders (partial or complete response) and non-responders (stable disease or progression).

We noted no differences in demographic characteristics, clinical data, and laboratory results (red cell distribution width (RDW) (*p* = 0.006) prior to therapy. Detailed characteristics of the patients within the analyzed groups are presented in Table 2.

### 3.1. The Multivariable Model for Therapy Response

In the third step, uni- and multivariable models for six months of therapy results (complete or partial response) were created, including demographic, clinical, and laboratory results.

Demographic characteristics—such as sex, age, and BMI—followed by clinical (arterial hypertension, diabetes mellitus, nicotine use, and family history positive for oncological diseases) were accompanied by surgical interventions prior to systemic therapy. All aforementioned parameters may have a significant impact on applied therapy. As there were two chemotherapeutic protocols, one was taken into consideration. The laboratory results (obtained before systemic therapy administration) were analysed, including peripheral blood count (hemoglobin and MLR, both presented in previous reports as possible biomarkers, followed by RDW, which was statistically different between both analyzed groups) and kidney function tests (for creatinine).

Predictive factors for six-month disease regression were established in univariable analysis. The multivariable model revealed RDW value prior to the applied systemic therapy (OR: 0.81, 95% CI: 0.65–1.00, *p* = 0.049) as a possible predictor of systemic treatment response in colorectal cancer, as presented in Table 3.

### 3.2. Receiver Operating Curve (ROC) for Disease Regression

**Figure 1:**
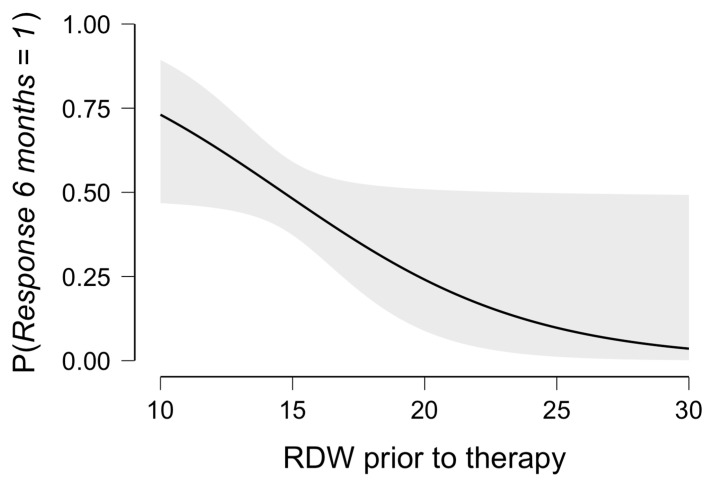
Predicted plot for therapy response related to RDW prior to chemotherapy administration.

The receiver operating characteristic curve revealed a predictive value of RDW for applied therapy with an area under the curve of 0.672, yielding a sensitivity of 70.0%, a specificity of 58.1%, and a precision of 0.609. The predicted estimates plot for RDW in response to therapy is presented in Figure 1.

## 4. Discussion

The results of our analysis indicate sex-related discrepancies in 6-month palliative chemotherapy outcomes based on CT scanning in colorectal cancer. The results suggest the possible predictive value of red cell distribution width from peripheral blood analysis for partial response to administered therapy.

It is postulated that doublet chemotherapy with targeting agents is the optimal therapeutic option in unresectable metastatic colorectal cancer patients [22].

It is worth noting that FOLFOX resistance in advanced CRC is significantly associated with upregulation and downregulation of several serum miRNAs, such as miR-19a. In terms of treatment response to anti-VEGF or anti-EGFR inhibitors in metastatic CRC, upregulation of miR-126 was correlated with bevacizumab resistance, whereas overexpression of miR-31, miR-100, and miR-125b and downregulation of miR-7 with resistance to cetuximab, respectively [23].

The possible impact of inflammatory activation on cancerogenesis, tumor progression, and treatment resistance is postulated. Immunological mechanisms and gut microbiota disturbances may trigger genetic alterations, ultimately leading to cancerogenesis [24]. In tumor progression, the interplay between inflammatory stimulation and the tumor microenvironment results in immune suppression, including the involvement of inflammasomes, cytokines, and non-coding RNAs [25]. The inflammatory response to applied therapy in oncological patients is considered a significant inherited resistance factor. In Baruch et al.’s study [26], tumors from non-responders were characterized by a constant interferon type I (IFN-I) signaling accompanied by higher M2 macrophage and T regulatory cell activity.

The efficacy of applied therapy depends on clinical factors and genetic analyses. In metastatic disease, systemic therapy remains the cornerstone of therapy. Strategies to improve survival and reduce disease progression are of the utmost importance. Identifying predictive markers in GIC metastatic cancer would enable personalized therapy and amended outcomes. Immune checkpoint inhibitors (ICIs) have given us a chance to ensure durable immune response, though they are used on a low number of patients [27].

Immune cell PD-L1 expression is significantly higher in mismatch repair (MMR)-deficient (MSI-H) CRC as compared to MMR-proficient (MSI-L) tumors, with no differences among the different MSI-H molecular subtypes. The recommended screening protocol for defective DNA MMR includes immunohistochemistry (IHC) and/or MSI testing. However, there are challenges in distilling the biological and technical heterogeneity of MSI testing down to usable data. It has been reported in the literature that IHC testing of the MMR machinery may produce different results for a given germline mutation, and it has been suggested that this may be due to somatic mutations [28].

The relationship between cancer disease and patients’ nutritional status has been postulated in previous studies [29]. The components of whole blood count have also been proposed as possible predictive markers [30,31].

The relationship between inflammatory activation and the development and progression of various malignancies, including colorectal cancer, has been postulated [32]. In chronic inflammatory bowel diseases, inflammatory-related tissue damage predisposes individuals to tumorigenesis, including CRC [33]. One of the most commonly available laboratory tests in clinical practice is peripheral blood analysis, which plays a significant role in the diagnosis of various diseases. There are numerous reports indicating the usefulness of hematology-associated inflammatory markers such as platelet-to-lymphocyte ratio (PLR), neutrophil-to-lymphocyte ratio (NLR), lymphocyte-to-monocyte ratio (LMR), lymphocyte-to-leukocyte ratio (LWR), neutrophil-to-monocyte ratio (NMR), and lymphocyte-to-CRP ratio (LCR) in colorectal cancer prognosis and therapy monitoring [34].

The red cell distribution width (RDW) describes the size heterogeneity of the circulating red blood cells. It is calculated by dividing the mean red cell volume (MCV) by the standard deviation of the red cell volume (SD) and multiplying by 100%. It has been postulated as an independent predictor of heart failure mortality [35]. The prognostic properties of RDW in cardiovascular and respiratory diseases have been noted in previous reports [36,37,38].

The possible utility of RDW results in colorectal cancer prognosis has been proposed in recent publications. An opposite relationship between RDW and CRC has been postulated, indicating that preoperative high RDW is a strong diagnostic [39] and poor prognostic marker [40], in contrast to Coradduzza et al.’s analysis [41]. Wen et al. [42] performed a meta-analysis on seven studies involving 7541 patients, and RDW was found to be predictive of overall survival and disease-free survival. The potential clinical utility of RDW in the anticipation of CRC patients’ outcomes was confirmed, in contrast to diagnostic characteristics. The higher RDW values were aligned with a right-sided CRC diagnosis by Fancellu et al. [43], but RDW’s diagnostic properties in CRC patients were not noticed in the retrospective analysis performed by Jiang et al. [44].

Our results suggest the possible value of higher RDW values obtained from peripheral blood counts taken before systemic chemotherapy as predictors of six-month disease progression risk. Our results indicate weak but significant discrimination, which, when incorporated with other factors, could be useful in improving palliative therapy results. Our study’s exact aim was to point out the possible factors related to disease regression or stabilization, indicating within the multivariable model the significance of lower red cell distribution width among patients with metastatic CRC. Further analyses are required, and the creation of a possible “risk score” for palliative CRC therapy could be considered in the future.

### Study Limitations

A retrospective analysis was performed on patients presenting with terminal digestive system cancers and metastatic spread. This study included various systemic therapeutic approaches and focused on first-line therapy in colorectal cancer in the terminal stage treated according to current guidelines [45].

Future studies involving large-scale prospective analyses are advisable to confirm the presented results. RDW could be incorporated into formation potential risk scores, which could help to improve CRC outcomes.

## 5. Conclusions

The results of our analysis point out the possible modulatory impact of red cell distribution width from peripheral blood analysis examined prior to systemic therapy on six-month results in colorectal terminal cancer management. Further prospective multicenter studies on a large population are required to confirm the presented results.

## Figures and Tables

**Table 1:** Patients’ demographic and clinical characteristics, followed by applied therapy, in relation to their response.

Parameters	Complete Response (1) n = 2	Partial Response (2) n = 38	Stable Disease (3) n = 36	Disease Progression (4) n = 7	P 1 vs. 2	P 1 vs. 3	P 1 vs. 4	P 2 vs. 3	p 2 vs. 4	P 3 vs. 4
Demographic										
Sex (M (%))	1 (50)	26 (68)	4 (57)	6 (60)	1.000	0.249	0.417	<0.001	0.488	0.745
Age (y) (median (Q1–Q3))	69 (68–70)	68 (62–73)	71 (65–72)	72 (69–77)	0.673	0.845	0.678	0.371	0.028	0.835
BMI (median (Q1–Q3))	26 (25–28)	26 (24–28)	25 (22–29)	25 (23–29)	0.901	0.782	0.802	0.793	0.732	0.836
Clinical										
AH (n (%))	2 (100)	21 (55)	23 (64)	5 (71)	0.499	0.538	1.000	0.486	0.174	0.294
DM (n (%))	0 (0)0	9 (23)	7 (19)	0 (0)	0.565	1.000	1.000	0.780	0.433	0.675
Nicotine (n (%))	0 (0)	7 (18)	5 (14)	0 (0)	0.688	1.000	1.000	0.438	0.477	0.714
FH (n (%))	1 (50)	8 (21)	6 (17)	4 (57)	0.401	0.339	1.000	0.769	0.131	0.489
Therapy					1.000	0.501	1.000	0.036	0.437	0.040
CTH (n (%))	1 (50)	18 (47)	26 (72)	2 (29)						
CTH-aEFGR (n (%))	1 (50)	20 (53)	10 (28)	5 (71)						
Metastases										
Multiple sides (n (%))	1(50)	14 (37)	14 (39)	0 (0)	1.000	0.498	0.222	1.000	0.081	0.081
including lungs (n (%))	0 (0)	12 (32)	12 (33)	3 (43)	1.000	1.000	0.500	1.000	0.670	0.680
including liver (n (%))	2 (100)	23 (61)	23 (64)	2 (29)	0.519	0.538	0.167	0.814	0.214	0.110
Surgery prior to therapy (n (%))	2 (100)	24 (63)	24 (67)	7 (100)	0.533	1.000	1.000	0.811	0.081	0.163

Abbreviations: CTH—systemic chemotherapy, CTH-aEFGR—systemic chemotherapy plus anti-epidermal growth factor receptor antibody, AH—arterial hypertension, BMI—body mass index, DM—diabetes mellitus, FH—positive oncological family history, kg—kilograms, M—male, m^2^—square meter, n—number, y—years.

**Table 2:** Patients’ demographic, clinical, and laboratory characteristics, followed by applied therapy in relation to response (complete or partial) vs. no response (stable disease or progression).

Parameters	Response Group n = 40	No Response Group n = 43	*p*
Demographic			
Sex (M (%)/F (%))	27 (63)/13 (37)	25 (63)/18 (27)	0.384
Age (years) (median (Q1–Q3)	68 (63–73)	70 (64–76)	0.190
BMI (kg/m^2^) (median (Q1–Q3)	26 (24–28)	26 (24–28)	0.678
Comorbidities			
Arterial hypertension (n (%))	23 (55)	28 (60)	0.482
Diabetes mellitus (n (%))	9 (25)	7 (19)	0.446
Nicotine (n (%))	7 (35)	5 (25)	0.194
Oncological family history (n (%))	9 (25)	10 (25)	0.928
Systemic therapy			0.109
CTH (n (%))	19 (48)	28 (65)	
CTH-aEFGR (n (%))	21 (52)	15 (35)	
Laboratory results prior to therapy			
WBC (109/dL) (median (Q1–Q3))	6.93 (5.55–8.74)	6.93 (5.43–8.57)	0.544
Lymphocyte (109/dL) (median (Q1–Q3))	1.39 (1.11–1.73)	1.36 (1.18–1.63)	0.913
Neutrophil (109/dL) (median (Q1–Q3))	4.52 (3.68–6.58)	4.52 (3.41–6.01)	0.678
Monocyte (109/dL) (median (Q1–Q3))	0.55 (0.40–0.67)	0.51 (0.43–0.67)	0.898
MLR (median (Q1–Q3))	0.39 (0.25–0.53)	0.41 (0.27–0.49)	0.888
Hb median (mmol/L) (median (Q1–Q3))	12.9 (11.7–13.9)	12.9 (11.6–13.5)	0.457
Hct (%) (median (Q1–Q3))	40 (37–42)	39 (35–42)	0.297
Plt (109/dL) (median (Q1–Q3))	249 (209–311)	266 (215–296)	0.719
RDW (%) (median (Q1–Q3))	13.8 (13.2–15.5)	14.9 (13.9–16.2)	0.007
Creatinine (mg/dL) (median (Q1–Q3))	0.9 (0.8–1.0)	0.9 (0.7–1.0)	0.975
AST (IU/L) (median (Q1–Q3))	22 (18–29)	21 (16–26)	0.167
Metastases			
Multiple sides (n (%))	16 ()	14 ()	0.487
including lungs (n (%))	12 ()	15 ()	0.641
including liver (n (%))	31 ()	25 ()	0.062
Surgery prior to therapy (n(%))	30	31 ()	0.770

Abbreviations: AST—aspartate transaminase, BMI—body mass index, CTH-aEFGR—systemic chemotherapy plus anti-epidermal growth factor receptor antibody, dL—decilitre, CTH—systemic chemotherapy, Hb—hemoglobin, Hct—hematocrit, IU—units, kg—kilograms, L—litre, M—male, mg—milligram, MLR—monocyte-to-lymphocyte ratio, m^2^—square meter, n–number, Plt—platelets, RDW—red cell distribution width, y—years, WBC—white blood count.

**Table 3:** Uni- and multivariable models for six-month therapy response in GIC patients.

Parameters	Univariable Model			Multivariable Model		
	OR	95% CI	*p*	OR	95% CI	*p*
Demographic						
Sex (male)	1.40	0.61–3.67	0.379			
Age	0.97	0.93–1.02	0.270			
BMI	1.03	0.44–3.09	0.395			
Clinical						
Arterial hypertension	0.73	0.30–1.80	0.477			
Diabetes mellitus	1.49	0.50–4.48	0.474			
Nicotine	1.91	0.57–6.44	0.297			
Oncological family history	0.98	0.34–2.81	0.963			
Surgery prior to systemic therapy	1.16	0.44–3.09	0.764			
Therapy						
CTH therapy	0.49	0.20–1.17	0.108			
Laboratory results prior to therapy				0.81	0.65–1.00	0.049
MLR	1.20	0.87–1.14	0.874			
RDW	0.80	0.61–1.00	0.040			
Hb	1.18	0.89–1.58	0.254			
Creatinine	0.67	0.08–5.50	0.701			
AST	1.01	0.99–1.03	0.431			

Abbreviations: AST—aspartate transaminase, BMI—body mass index, CI—confidence interval, CTH—chemotherapy, Hb—hemoglobin, MLR—monocyte-to-lymphocyte ratio, OR—odds ratio, RDW—red cell distribution width.

## Data Availability

Data supporting the presented results will be available upon reasonable request to the corresponding author for three years after publication.

## Abbreviations

The following abbreviations are used in this manuscript:

a-EGFR	anti-epidermal growth factor receptor
AH	arterial hypertension
AST	aspartate aminotransferase,
BMI	body mass index
CI	confidence interval
CRC	colorectal cancer
CT	computed tomography
CTH	chemotherapy
DM	diabetes mellitus
FH	positive family history for oncological disease
GIC	gastrointestinal cancer
Hct	hematocrit
kg	kilograms
M	male
MLR	monocyte-to-lymphocyte ratio
m^2^	square meter
n	number
OR	odds ratio
Plt	platelets
RDW	red cell distribution width
ROC	receiver operating curve
y	years
Wbc	white blood count

## References

[1] Arnold M., Abnet C.C., Neale R.E., Vignat J., Giovannucci E.L., McGlynn K.A., Bray F. (2020). Global Burden of 5 Major Types of Gastrointestinal Cancer. Gastroenterology.

[2] Danpanichkul P., Suparan K., Tothanarungroj P., Dejvajara D., Rakwong K., Pang Y., Barba R., Thongpiya J., Fallon M.B., Harnois D. (2024). Epidemiology of gastrointestinal cancers: A systematic analysis from the Global Burden of Disease Study 2021. Gut.

[3] Wang S., Zheng R., Li J., Zeng H., Li L., Chen R., Sun K., Han B., Bray F., Wei W. (2024). Global, regional, and national lifetime risks of developing and dying from gastrointestinal cancers in 185 countries: A population-based systematic analysis of GLOBOCAN. Lancet Gastroenterol. Hepatol..

[4] Mysuru Shivanna L., Urooj A. (2016). A Review on Dietary and Non-Dietary Risk Factors Associated with Gastrointestinal Cancer. J. Gastrointest. Cancer.

[5] Baidoun F., Elshiwy K., Elkeraie Y., Merjaneh Z., Khoudari G., Sarmini M.T., Gad M., Al-Husseini M., Saad A. (2021). Colorectal Cancer Epidemiology: Recent Trends and Impact on Outcomes. Curr. Drug Targets.

[6] Carbone F., Spinelli A., Ciardiello D., Realis Luc M., de Pascale S., Bertani E., Fazio N., Fumagalli Romario U. (2025). Prognosis of early-onset versus late-onset sporadic colorectal cancer: Systematic review and meta-analysis. Eur. J. Cancer.

[7] Sninsky J.A., Shore B.M., Lupu G.V., Crockett S.D. (2022). Risk Factors for Colorectal Polyps and Cancer. Gastrointest. Endosc. Clin. N. Am..

[8] Morgan E., Arnold M., Gini A., Lorenzoni V., Cabasag C.J., Laversanne M., Vignat J., Ferlay J., Murphy N., Bray F. (2023). Global burden of colorectal cancer in 2020 and 2040: Incidence and mortality estimates from GLOBOCAN. Gut.

[9] Osseis M., Nehmeh W.A., Rassy N., Derienne J., Noun R., Salloum C., Rassy E., Boussios S., Azoulay D. (2022). Surgery for T4 Colorectal Cancer in Older Patients: Determinants of Outcomes. J. Pers. Med..

[10] Rawla P., Sunkara T., Barsouk A. (2019). Epidemiology of colorectal cancer: Incidence, mortality, survival, and risk factors. Prz. Gastroenterol..

[11] Zhao J., Wang Q., Tan A.F., Loh C.J.L., Toh H.C. (2024). Sex differences in cancer and immunotherapy outcomes: The role of androgen receptor. Front. Immunol..

[12] Dufrusine B., Di Lisio C., Maurizio A., Sallese M., De Laurenzi V., Dainese E. (2023). Influence of food emulsifiers on cellular function and inflammation, a preliminary study. Front. Nutr..

[13] Yang H., Wang X.K., Wang J.B., Zhao F.H., Fan J.H., Qiao Y.L., Taylor P.R., Abnet C.C. (2022). Combined risk factors and risk of upper gastrointestinal cancer mortality in the Linxian general population. Int. J. Cancer.

[14] Grady W.M., Yu M., Markowitz S.D. (2021). Epigenetic Alterations in the Gastrointestinal Tract: Current and Emerging Use for Biomarkers of Cancer. Gastroenterology.

[15] Yamamoto T., Kawada K., Obama K. (2021). Inflammation-Related Biomarkers for the Prediction of Prognosis in Colorectal Cancer Patients. Int. J. Mol. Sci..

[16] Lin N., Li J., Yao X., Zhang X., Liu G., Zhang Z., Weng S. (2022). Prognostic value of neutrophil-to-lymphocyte ratio in colorectal cancer liver metastasis: A meta-analysis of results from multivariate analysis. Int. J. Surg..

[17] Fuss J., Voloboyeva A., Polovyj V., Yaremkevych R. (2022). Neutrophil to lymphocyte ratio in predicting postoperative complications and prognosis in patients with colorectal cancer. Pol. Prz. Chir..

[18] Chen Q., Li G.L., Zhu H.Q., Yu J.D., Chen Z.P., Wu J.Y., Lin Z.Y., Wan Y.L. (2023). The neutrophil-to-lymphocyte ratio and lactate dehydrogenase combined in predicting liver metastasis and prognosis of colorectal cancer. Front. Med..

[19] Naito Y., Nishida T., Doi T. (2023). Current status of and future prospects for the treatment of unresectable or metastatic gastrointestinal stromal tumours. Gastric Cancer.

[20] Lote H., Chau I. (2024). Immunotherapy in Gastrointestinal Cancers. Cancer Treat. Res..

[21] Chong X., Madeti Y., Cai J., Li W., Cong L., Lu J., Mo L., Liu H., He S., Yu C. (2024). Recent developments in immunotherapy for gastrointestinal tract cancers. J. Hematol. Oncol..

[22] Kim J.H., Cha Y., Shin S.J., Park Y.S., Kang J.H., Kim C., Lim S.H., Kang M.J., Kim J.G., Hwang I.G. (2024). Treatment Patterns and Prognosis of Palliative Chemotherapy Combined with Targeting Agents in Patients with Unresectable Metastatic Colorectal Cancer: CHOICE, A Multicenter Longitudinal Observational Study. Anticancer Res..

[23] Koncina E., Haan S., Rauh S., Letellier E. (2020). Prognostic and Predictive Molecular Biomarkers for Colorectal Cancer: Updates and Challenges. Cancers.

[24] Vanoli A., Parente P., Fassan M., Mastracci L., Grillo F. (2023). Gut inflammation and tumorigenesis: Every site has a different tale to tell. Intern. Emerg. Med..

[25] Denk D., Greten F.R. (2022). Inflammation: The incubator of the tumor microenvironment. Trends Cancer.

[26] Baruch E.N., Nagarajan P., Gleber-Netto F.O., Rao X., Xie T., Akhter S., Adewale A., Shajedul I., Mattson B.J., Ferrarotto R. (2023). Inflammation induced by tumor-associated nerves promotes resistance to anti-PD-1 therapy in cancer patients and is targetable by interleukin-6 blockade. Biol. Sci..

[27] Santini D., Zeppola T., Russano M., Citarella F., Anesi C., Buti S., Tucci M., Russo A., Sergi M.C., Adamo V. (2021). PD-1/PD-L1 checkpoint inhibitors during late stages of life: An ad-hoc analysis from a large multicenter cohort. J. Transl. Med..

[28] Chang L., Chang M., Chang H.M., Chang F. (2017). Expending Role of Microsatellite Instability in Diagnosis and Treatment of Colorectal Cancers. J. Gastrointest. Cancer.

[29] Park S.H., Lee S., Song J.H., Choi S., Cho M., Kwon I.G., Son T., Kim H.I., Cheong J.H., Hyung W.J. (2020). Prognostic significance of body mass index and prognostic nutritional index in stage II/III gastric cancer. Eur. J. Surg. Oncol..

[30] Qi W.X., Wang X., Li C., Li S., Li H., Xu F., Chen J., Zhao S., Li H. (2023). Pretreatment absolute lymphocyte count is an independent predictor for survival outcomes for esophageal squamous cell carcinoma patients treated with neoadjuvant chemoradiotherapy and pembrolizumab: An analysis from a prospective cohort. Thorac. Cancer.

[31] Wu Y., Chen J., Zhao L., Li Q., Zhu J., Yang H., Guo S., Xi M. (2021). Prediction of Pathologic Response to Neoadjuvant Chemoradiotherapy in Patients with Esophageal Squamous Cell Carcinoma Incorporating Hematological Biomarkers. Cancer Res. Treat..

[32] Koržinek M., Ćelap I., Fabijanec M., Žanić T., Ljubičić N., Baršić N., Verbanac D., Barišić K., Rajković M.G. (2025). Complete blood count parameters and inflammation-related biomarkers in patients with colorectal carcinoma. Acta Pharm..

[33] Zhao H., Wu L., Yan G., Chen Y., Zhou M., Wu Y., Li Y. (2021). Inflammation and tumor progression: Signaling pathways and targeted intervention. Signal Transduct. Target. Ther..

[34] Virdee P.S., Marian I.R., Mansouri A., Elhussein L., Kirtley S., Holt T., Birks J. (2020). The Full Blood Count Blood Test for Colorectal Cancer Detection: A Systematic Review, Meta-Analysis, and Critical Appraisal. Cancers.

[35] Felker G.M., Allen L.A., Pocock S.J., Shaw L.K., McMurray J.J., Pfeffer M.A., Swedberg K., Wang D., Yusuf S., Michelson E.L. (2007). Red cell distribution width as a novel prognostic marker in heart failure: Data from the CHARM Program and the Duke Databank. J. Am. Coll. Cardiol..

[36] Sobieraj M., Urbanowicz T., Olasińska-Wiśniewska A., Gładki M., Michalak M., Filipiak K.J., Węclewska A., Bartkowska-Śniatkowska A., Tykarski A., Bobkowski W. (2024). Anisocytosis as a possible predictor of low cardiac output syndrome in children undergoing mitral valve surgery. Adv. Med. Sci..

[37] Fava C., Cattazzo F., Hu Z.D., Lippi G., Montagnana M. (2019). The role of red blood cell distribution width (RDW) in cardiovascular risk assessment: Useful or hype?. Ann. Transl. Med..

[38] He Q., Hu S., Xie J., Liu H., Li C. (2024). The red blood cell distribution width to albumin ratio was a potential prognostic biomarker for acute respiratory failure: A retrospective study. BMC Med. Inform. Decis. Mak..

[39] Song Y., Huang Z., Kang Y., Lin Z., Lu P., Lin Q., Cai Z., Cao Y., Zhu X. (2018). Clinical Usefulness and Prognostic Value of Red Cell Distribution Width in Colorectal Cancer. BioMed Res. Int..

[40] Li Y., Xing C., Wei M., Wu H., Hu X., Li S., Sun G., Zhang G., Wu B., Zhang F. (2019). Combining Red Blood Cell Distribution Width (RDW-CV) and CEA Predict Poor Prognosis for Survival Outcomes in Colorectal Cancer. J. Cancer.

[41] Coradduzza D., Medici S., Chessa C., Zinellu A., Madonia M., Angius A., Carru C., De Miglio M.R. (2023). Assessing the Predictive Power of the Hemoglobin/Red Cell Distribution Width Ratio in Cancer: A Systematic Review and Future Directions. Medicina.

[42] Wen Z.L., Zhou X., Xiao D.C. (2022). Is red blood cell distribution width a prognostic factor for colorectal cancer? A meta-analysis. Front. Surg..

[43] Fancellu A., Zinellu A., Mangoni A.A., Popova A., Galotti F., Feo C.F., Attene F., Cossu A., Palmieri G., Paliogiannis P. (2022). Red Blood Cell Distribution Width (RDW) Correlates to the Anatomical Location of Colorectal Cancer. Implications for Clinical Use. J. Gastrointest. Cancer.

[44] Jiang C., Huang J.H., Cong H. (2024). Clinical Value of Routine Biomarkers for Colorectal Patients: A Retrospective Study. Clin. Lab..

[45] Casali P.G., Blay J.Y., Abecassis N., Bajpai J., Bauer S., Biagini R., Bielack S., Bonvalot S., Boukovinas I., Bovee J.V.M.G. (2022). Gastrointestinal stromal tumours: ESMO-EURACAN-GENTURIS Clinical Practice Guidelines for diagnosis, treatment and follow-up. Ann. Oncol..

